# Associations Between Schizophrenia Polygenic Liability, Symptom Dimensions, and Cognitive Ability in Schizophrenia

**DOI:** 10.1001/jamapsychiatry.2021.1961

**Published:** 2021-08-04

**Authors:** Sophie E. Legge, Alastair G. Cardno, Judith Allardyce, Charlotte Dennison, Leon Hubbard, Antonio F. Pardiñas, Alexander Richards, Elliott Rees, Arianna Di Florio, Valentina Escott-Price, Stanley Zammit, Peter Holmans, Michael J. Owen, Michael C. O’Donovan, James T. R. Walters

**Affiliations:** 1 MRC Centre for Neuropsychiatric Genetics and Genomics, Division of Psychological Medicine and Clinical Neurosciences, Cardiff University School of Medicine, Cardiff, United Kingdom; 2 Leeds Institute of Health Sciences, Division of Psychological and Social Medicine, Faculty of Medicine and Health, University of Leeds, Leeds, United Kingdom; 3 Centre for Clinical Brain Sciences, University of Edinburgh, Edinburgh, United Kingdom; 4 Centre for Academic Mental Health, Department of Population Health Sciences, Bristol Medical School, University of Bristol, Bristol, United Kingdom

## Abstract

**Importance:**

Schizophrenia is a clinically heterogeneous disorder. It is currently unclear how variability in symptom dimensions and cognitive ability is associated with genetic liability for schizophrenia.

**Objective:**

To determine whether phenotypic dimensions within schizophrenia are associated with genetic liability to schizophrenia, other neuropsychiatric disorders, and intelligence.

**Design, Setting, and Participants:**

In a genetic association study, 3 cross-sectional samples of 1220 individuals with a diagnosis of schizophrenia were recruited from community, inpatient, and voluntary sector mental health services across the UK. Confirmatory factor analysis was used to create phenotypic dimensions from lifetime ratings of the Scale for the Assessment of Positive Symptoms, Scale for the Assessment of Negative Symptoms, and the MATRICS Consensus Cognitive Battery. Analyses of polygenic risk scores (PRSs) were used to assess whether genetic liability to schizophrenia, other neuropsychiatric disorders, and intelligence were associated with these phenotypic dimensions. Data collection for the cross-sectional studies occurred between 1993 and 2016. Data analysis for this study occurred between January 2019 and March 2021.

**Main Outcomes and Measures:**

Outcome measures included phenotypic dimensions defined from confirmatory factor analysis relating to positive symptoms, negative symptoms of diminished expressivity, negative symptoms of motivation and pleasure, disorganized symptoms, and current cognitive ability. Exposure measures included PRSs for schizophrenia, bipolar disorder, major depression, attention-deficit/hyperactivity disorder, autism spectrum disorder, and intelligence.

**Results:**

Of the 1220 study participants, 817 were men (67.0%). Participants’ mean (SD) age at interview was 43.10 (12.74) years. Schizophrenia PRS was associated with increased disorganized symptom dimension scores in both a 5-factor model (β = 0.14; 95% CI, 0.07-0.22; *P* = 2.80 × 10^−4^) and a 3-factor model across all samples (β = 0.10; 95% CI, 0.05-0.15; *P* = 2.80 × 10^−4^). Current cognitive ability was associated with genetic liability to schizophrenia (β = −0.11; 95% CI, −0.19 to −0.04; *P* = 1.63 × 10^−3^) and intelligence (β = 0.23; 95% CI, 0.16-0.30; *P* = 1.52 × 10^−10^). After controlling for estimated premorbid IQ, current cognitive performance was associated with schizophrenia PRS (β = −0.08; 95% CI, −0.14 to −0.02; *P* = 8.50 × 10^−3^) but not intelligence PRS.

**Conclusions and Relevance:**

The findings of this study suggest that genetic liability for schizophrenia is associated with higher disorganized dimension scores but not other symptom dimensions. Cognitive performance in schizophrenia appears to reflect distinct contributions from genetic liabilities to both intelligence and schizophrenia.

## Key Points

**Question:**

Are phenotypic dimensions in schizophrenia associated with genetic liability to schizophrenia, other neuropsychiatric disorders, and intelligence?

**Findings:**

In this cross-sectional genetic association study of 1220 individuals with schizophrenia, analyses indicated that higher levels of disorganized symptoms, but not other symptom dimensions, and lower levels of current cognitive ability were significantly associated with schizophrenia polygenic risk scores. Current cognitive ability was also associated with intelligence polygenic risk scores.

**Meaning:**

The findings of this study suggest that variation in disorganized symptoms and cognitive ability in schizophrenia are markers of schizophrenia common genetic liability; cognitive performance in schizophrenia may reflect distinct contributions from genetic liabilities to both intelligence and schizophrenia.

## Teaser

This genetic association study examines the associations between phenotypic dimensions in schizophrenia and genetic liability to schizophrenia, other neuropsychiatric disorders, and intelligence in patients with schizophrenia.

## Introduction

The clinical heterogeneity of schizophrenia and related psychotic disorders has long been recognized. Factor analyses of symptoms related to schizophrenia have consistently resulted in positive, negative, and disorganized dimensions.^1,2^ Studies have also identified dimensions relating to cognitive ability, which, although not considered a core diagnostic feature, has been consistently shown to be impaired in schizophrenia.^3,4^ A better understanding of the different factors underlying these symptom dimensions could contribute to the development of novel treatments for schizophrenia; currently available treatments predominantly affect only positive symptoms.

The genetic contribution to clinical variation between patients across symptom dimensions has been of interest for some time. Familial aggregation for a disorganized symptom dimension has been found in studies of affected sib-pairs^5,6^ and multigeneration families,^7^ although twin-based analysis has indicated that this aggregation is substantially a genetic effect (heritability; h^2^ = 84%).^1^ The disorganized dimension also demonstrates patterns of familial aggregation consistent with it being a marker of genetic loading for psychotic disorders.^2^ Evidence for familial aggregation of the positive and negative dimensions is less consistent, but the negative dimension has also been associated with genetic loading for psychotic disorders.^8^

Recent genomic studies have used polygenic risk scores (PRSs) to assess the association between schizophrenia genetic liability and phenotypic dimensions. The schizophrenia PRS has been reported to be associated with a combined disorganized/negative dimension^9,10^ and with negative symptoms.^4,11,12^ However, many of these studies have not assessed disorganized symptoms or are based on a dimensional structure that combines disorganized symptoms with either negative symptoms^9,10,13^ or cognition.^3^ Furthermore, there is emerging evidence that indicates the structure of negative symptoms may not be unidimensional and that at least 2 substructures can be reliably identified: diminished expressivity and diminished motivation and pleasure.^14,15^ The association of genetic liability with other psychiatric disorders on symptom dimensions in schizophrenia is unclear.

Cognitive ability in the general population is a highly heritable trait.^16^ However, studies investigating the genetic source of the cognitive deficits observed in individuals with schizophrenia and the association with schizophrenia genetic liability have reported inconsistent findings.^17,18,19^

In this genetic association study, we aimed to examine which phenotypic dimensions of schizophrenia are associated with genetic liability to schizophrenia as indexed by PRSs. We addressed gaps in previous studies by defining each of the commonly found symptom dimensions and cognitive ability as distinct phenotypic dimensions and conducted a meta-analysis across 3 samples that used a common approach to phenotypic assessment. We also investigated the association between phenotypic dimensions and genetic liability to psychiatric and cognitive traits other than schizophrenia.

## Methods

### Participants

This genetic association study included a total of 1220 individuals with schizophrenia. Participants came from 3 schizophrenia sample collections, all previously reported: the CardiffCOGS (n = 697),^20^ Cardiff F-series (n = 383),^21^ and Cardiff Affected-Sib (n = 140) samples^5^ (eTable 1 in the Supplement provides demographic characteristics). All studies had the relevant UK National Health Service ethical approval, and written informed consent was obtained for all study participants. All participants underwent a comprehensive clinical research interview based on the Schedules for Clinical Assessment in Neuropsychiatry^22^ and met *Diagnostic and Statistical Manual of Mental Disorders (Fourth Edition)*^23^ or *International Statistical Classification of Diseases and Related Health Problems, 10th Revision*^24^ criteria for a diagnosis of schizophrenia or schizoaffective disorder, depressed type. Recruitment to each study was from community, inpatient, and voluntary sector mental health services across the UK. The Cardiff Affected-Sib sample included a single affected individual from families with 2 or more siblings diagnosed with schizophrenia. The study was conducted at the MRC Centre for Neuropsychiatric Genetics and Genomics at Cardiff University, UK. Data collection for the cross-sectional studies occurred between 1993 and 2016. Data analysis for this study occurred between January 2019 and March 2021. This study followed the Strengthening the Reporting of Genetic Association Studies (STREGA) reporting guideline.

### Phenotype Measures

In each of the 3 samples, the Scale for the Assessment of Positive Symptoms^25^ and the Scale for the Assessment of Negative Symptoms^26^ were scored on a lifetime worst basis using information from the Schedules for Clinical Assessment in Neuropsychiatry interview and lifetime psychiatric clinical case notes. Measures of cognition were available in the CardiffCOGS sample only: we used the MATRICS Consensus Cognitive Battery domain and composite scores^27^ as a measure of current cognitive ability and the National Adult Reading Test^28^ as a measure of estimated premorbid IQ. eTable 2 in the Supplement details the phenotype measures used.

Trained psychiatrists or psychology graduates completed the clinical ratings and cognitive assessments under the supervision of study principal investigators (consultant psychiatrists) (A.G.C., S.Z., M.J.O., M.C.O., and J.T.R.W.), and regular interrater reliability was measured. The Scale for the Assessment of Positive Symptoms and Scale for the Assessment of Negative Symptoms ratings had good interrater reliability in each sample, with κ values ranging from 0.72 to 0.95. Interrater reliability for research diagnosis was described in a previous publication.^29^

### Genetic Data

All samples were genotyped on the Illumina HumanOmniExpress (version 8 or 12). Details relating the quality control and imputation of genetic data are provided in eAppendix 1 in the Supplement. Genetic principal components representing ancestry were derived using PLINK, version 2.0^30^ using single-nucleotide variants with low levels of linkage disequilibrium (*r*^2^ <0.2 and 500-kilobyte window; criteria used by the Psychiatric Genomics Consortium^31^). All genetic analyses were restricted to individuals of European ancestry as assessed by principal components. Our sample contained too few participants of non-European ancestry to analyze. First-degree relatives (π >0.4) both within and between samples were identified, and 1 member of each related pair was removed, preferentially retaining samples that had more complete phenotype data and otherwise removed at random.

### Polygenic Risk Scores

Polygenic risk scores were calculated using PRSice^32^ following a widely applied method^33^ and using default parameters unless otherwise stated. Criteria used by the Psychiatric Genomics Consortium^31^ were applied to select common single-nucleotide variants (minor allele frequency, >0.10) of high-quality (imputation score, >0.9) in relative linkage equilibrium (*r*^2^ <0.2, 500-kilobyte window) that were present in all 3 samples. We excluded the extended major histocompatibility complex region (25-34 megabytes) given its complex linkage disequilibrium structure. The PRSs for schizophrenia were calculated for each study participant using summary statistics from the largest available genome-wide association study^34^ after excluding the samples included in this study (summary statistics derived specifically for the purposes of this study by the Psychiatric Genomics Consortium Schizophrenia Working Group). Scores were also calculated using the largest available genome-wide association study for bipolar disorder,^35^ major depression,^36^ autism spectrum disorder,^37^ attention-deficit/hyperactivity disorder (ADHD),^38^ and intelligence.^16^ The first 5 genetic principal components were included as covariates when generating each PRS. We selected the PRSs based on single-nucleotide variants associated with a threshold of *P* ≤ .05 in the genome-wide association study sample for the primary analysis given that single-nucleotide variant inclusion at this threshold gives the best prediction of schizophrenia,^31^ but as a secondary sensitivity test, we also looked across a range of *P* value thresholds: 5 × 10^−8^, 1 × 10^−7^, 1 × 10^−6^, 1 × 10^−5^, 1 × 10^−4^, 0.001, 0.05, 0.1, 0.2, 0.5, and 1.0.

### Statistical Analysis

A confirmatory factor analysis (CFA) framework was used to estimate phenotype-derived dimension scores. First, CFA of symptom and cognition measures was conducted on the CardiffCOGS sample because CardiffCOGS had comprehensive ratings for cognition and other symptoms not measured in the other samples. Next, we fitted a multiple-group CFA model solely of symptom ratings that were available across all 3 samples.

The lavaan package^39^ for R was used to identify all CFA models. A series of prespecified models were examined in the CardiffCOGS sample (detailed in eTable 2 in the Supplement) using empirically derived and validated measurement models from previous publications.^5,14^ The Scale for the Assessment of Positive Symptoms and Scale for the Assessment of Negative Symptoms measures were global scores of symptom categories, with the exception of inappropriate affect, and were included on an ordinal (0-5) scale in the CFA models. All MATRICS Consensus Cognitive Battery domain scores were included on a continuous scale. Otherwise, we used default parameters of the lavaan package, including fixing the first indicator variable to 1, automatically adding residual variances, and allowing latent variables to be correlated.

The model that was considered to have the best fit was selected for further analyses. Model fit was guided by both theoretical knowledge and standard interpretations of goodness-of-fit indices,^40,41^ including (1) the standardized root mean square residual (<0.08), (2) the root mean square error of approximation (<0.06), and (3) the comparative fit index (>0.95). eAppendix 2 in the Supplement provides further details of goodness-of-fit indices. Phenotype dimension scores were calculated for each study participant from the CFA model.

Polygenic risk scores and phenotype dimension scores were standardized before analysis. Each phenotype dimension (considered the dependent variable) was regressed on the schizophrenia PRS using linear regression and including sex, age at interview, and the first 5 genetic principal components as covariates. For the multisample analysis, β values were meta-analyzed using the R package meta,^42^ with a fixed-effect model weighted by the SE. To control for multiple testing, a Bonferroni correction was applied for the number of phenotype dimensions tested. In secondary analyses, we also tested the association of PRSs for bipolar disorder, depression, ADHD, autism spectrum disorder, and intelligence with each phenotype dimension. A Bonferroni correction was applied for testing 5 disorder PRSs multiplied by the number of phenotype dimensions. In CardiffCOGS, we also tested the correlation of estimated premorbid IQ with the derived cognitive dimension via Pearson correlation and assessed the association between premorbid IQ and schizophrenia PRS via linear regression.

## Results

A total of 1220 individuals were included in the study; 817 were men (67.0%), 403 were women (33.0%), and the mean (SD) age at study interview was 43.10 (12.74) years. eTable 1 in the Supplement provides demographic information specific to each sample.

**Figure 1.**
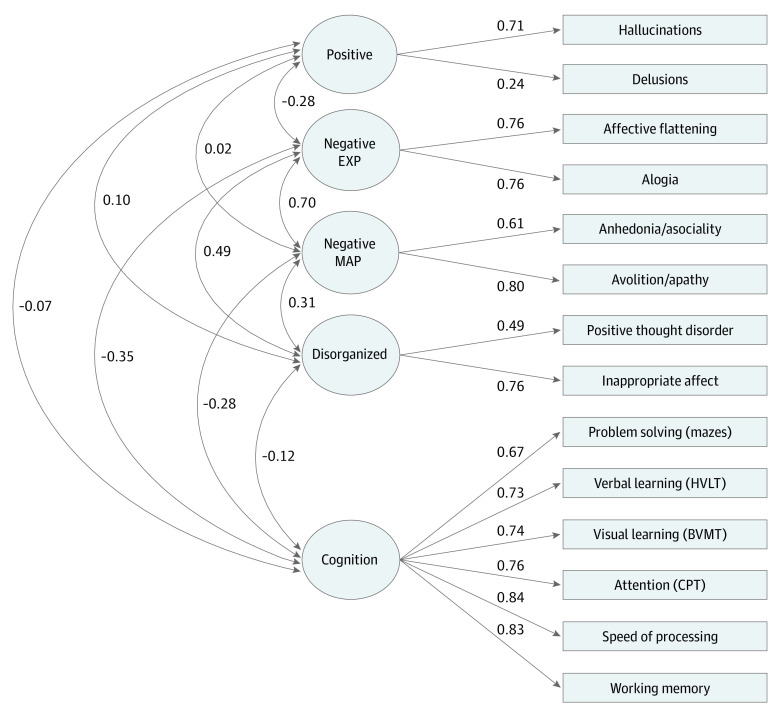
Five-Factor Structure of Phenotype Dimensions in Schizophrenia Structure of 5-factor phenotype dimensions derived by confirmatory factor analysis (CFA) in the CardiffCOGS sample. Boxes on the right detail contributing phenotypes, and circles represent latent factors created by CFA. Solid lines represent standardized factor loadings, and curved lines represent the correlation among phenotype dimensions. BVMT indicates Brief Visuospatial Memory Test–Revised; CPT, Continuous Performance Test: Identical Pairs; negative EXP, negative symptoms of diminished expressivity; HVLT, Hopkins Verbal Learning Test; and negative MAP, negative symptoms of diminished motivation and pleasure.

The CFA models fitted in the CardiffCOGS sample are detailed in eTable 2 in the Supplement. The optimal model had 5 phenotypic dimensions relating to positive symptoms, negative symptoms of diminished expressivity, negative symptoms of motivation and pleasure, disorganized symptoms, and cognitive ability. Figure 1 details the factor loadings of contributing phenotypes from the optimal model, which fitted the data well at the global level: comparative fit index, 0.99; root mean square error of approximation, 0.04; 95% CI, 0.03-0.05; and standardized root mean square residual, 0.05.

A 3-factor CFA model was fitted across all 3 samples for positive symptoms, negative symptoms of diminished expressivity, and disorganized symptoms. The 3-factor model was a good fit for the data in each sample; therefore no further post hoc modifications to the model were made (eFigure 1 in the Supplement details factor loadings and fit measures). The CFA dimension scores derived from the 5-factor and 3-factor models were highly correlated (0.92-0.98).

### Genetic Liability

**Figure 2.**
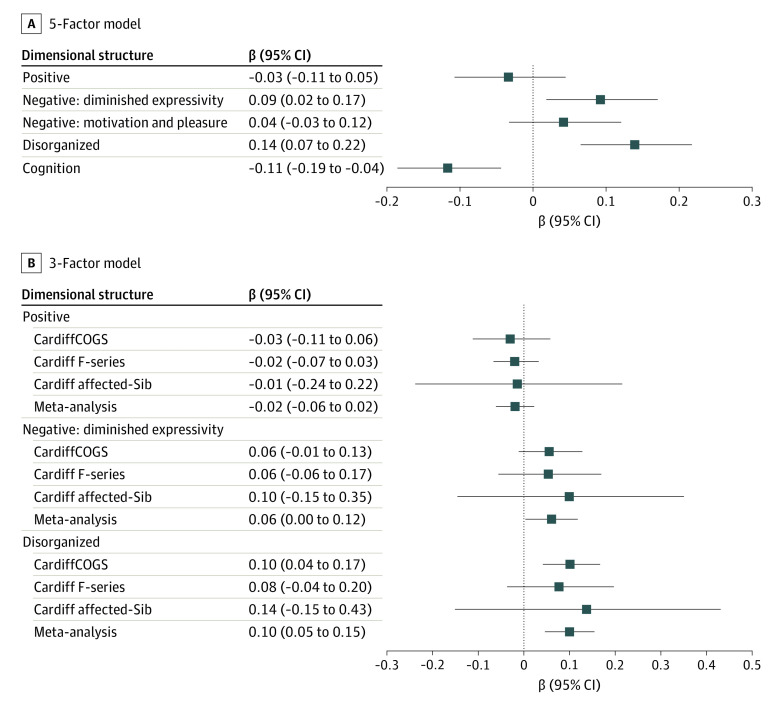
Association of Schizophrenia Polygenic Risk Score and Phenotype Dimensions Association of schizophrenia polygenic risk scores with phenotype dimensions. A, The association (β) with phenotypes derived in the 5-factor model in the CardiffCOGS sample. B, The association (β) with phenotypes derived in the 3-factor model for each of the 3 contributing samples and combined meta-analysis. All error bars represent the 95% CI of the β value. Dotted line represents a null model (values >0 indicate increased risk and values <0 indicate reduced risk).

Table 1 and Figure 2 detail the associations between schizophrenia PRS and the phenotype dimensions from the CardiffCOGS 5-factor model. Schizophrenia PRS was significantly associated with higher scores on the disorganized dimension (β = 0.14; 95% CI, 0.07-0.22; *P* = 2.80 × 10^−4^) and lower current cognitive ability (β= −0.11; 95% CI, −0.19 to −0.04; *P* = 1.63 × 10^−3^). The dimension of negative symptoms of diminished expressivity was associated at *P* < .05 (β = 0.09; 95% CI, 0.02-0.17; *P* = .015) but did not survive a Bonferroni correction for the 5 dimensions tested (*P* < .01). These associations were consistent across different *P* thresholds for single-nucleotide variant inclusion in the PRS (eTable 3 in the Supplement), but as expected, the optimal threshold was our primary *P* threshold of .05, which typically captures maximum heritability. To assess whether the phenotypic dimensions were independently associated with the PRS, we regressed the schizophrenia PRS against all phenotype dimensions simultaneously. In this model, the dimensions relating to disorganized symptoms and current cognitive ability remained significantly associated with schizophrenia PRS, whereas the dimension of negative symptoms of diminished expressivity did not (eTable 4 in the Supplement).

**Table 1.** Association of Schizophrenia PRS and Phenotype Dimensions From 5-Factor Model

Phenotype dimension	CardiffCOGS (n = 662)			
	β (95% CI)	SE	*R* ^2^	*P* value^a^
Positive	−0.03 (−0.11 to 0.05)	0.039	<0.001	.422
Negative				
Diminished expressivity	0.09 (0.02 to 0.17)	0.039	0.007	.015
Motivation and pleasure	0.04 (−0.03 to 0.12)	0.039	<0.001	.258
Disorganized	0.14 (0.07 to 0.22)	0.039	0.019	2.80 × 10^−4^
Cognition	−0.11 (−0.19 to −0.04)	0.036	0.012	1.63 × 10^−3^

Abbreviation: PRS, polygenic risk score.

^a^ Determined with regression analyses.

To confirm the findings applied to raw symptoms scores, we tested the association between schizophrenia PRS and summed raw phenotype scores used to create the symptom dimensions and the MATRICS Consensus Cognitive Battery composite score for cognition, as opposed to the latent factors defined by CFA, and found that schizophrenia PRS was associated only with disorganized symptoms (β = 0.24; 95% CI, 0.11-0.37; *P* = 2.12 × 10^−4^) and current cognitive ability (β = −0.13; 95% CI, −0.23 to −0.04; *P* = 4.63 × 10^−3^) (eFigure 2, eTable 5 in the Supplement). Schizophrenia PRS was associated with both of the variables used to create the disorganized dimension when entered into the same model simultaneously: positive formal thought disorder (β = 0.10; 95% CI, 0.02-0.18; *P* = .01) and inappropriate affect (β = 0.08; 95% CI, 0.01-0.16; *P* = .04). Schizophrenia PRS remained associated with the disorganized and cognitive dimensions when controlling for potential confounders of age at the onset of psychosis and treatment resistance to antipsychotics (eTable 6 in the Supplement).

In a meta-analysis of the multisample 3-factor model (Table 2, Figure 2B), schizophrenia PRS was significantly associated with the disorganized dimension (β = 0.10; 95% CI, 0.05-0.15; *P* = 2.80 × 10^−4^) after correction for multiple testing, but not with the dimensions relating to negative symptoms of diminished expressivity (β = 0.06; 95% CI, 0.00-0.12; *P* = .038) or positive symptoms (β = −0.02; 95% CI, −0.06 to 0.02; *P* = .367). Tests for heterogeneity (Cochran *Q* and *I*^2^) indicated that the studies in the meta-analysis had consistent outcomes for all phenotype dimensions studied.

**Table 2.** Meta-analysis of Schizophrenia PRS and Phenotype Dimensions From Multisample 3-Factor Model

Symptom dimension	CardiffCOGS (n = 697)^a^			Cardiff F-series (n = 383)			Cardiff affected-sib (n = 140)			Meta-analysis (n = 1220)			
	β (95% CI)	SE	*P* value	β (95% CI)	SE	*P* value	β (95% CI)	SE	*P* value	β (95% CI)	*P* value	Q	Q *P* value
Positive	−0.03 (−0.11 to 0.06)	0.043	.533	−0.02 (−0.07 to 0.03)	0.025	.501	−0.01 (−0.24 to 0.22)	0.114	.922	−0.02 (−0.06 to 0.02)	.367	0.04	.978
Negative: diminished expressivity	0.06 (−0.01 to 0.13)	0.035	.098	0.06 (−0.06 to 0.17)	0.057	.321	0.10 (−0.15 to 0.35)	0.125	.414	0.06 (0.00 to 0.12)	.038	0.12	.942
Disorganized	0.10 (0.04 to 0.17)	0.032	1.14×10^−3^	0.08 (−0.04 to 0.20)	0.059	.179	0.14 (−0.15 to 0.43)	0.147	.342	0.10 (0.05 to 0.15)	2.80×10^−4^	0.20	.903

Abbreviation: PRS, polygenic risk score.

^a^ The sample size for CardiffCOGS is larger than in the 5-factor model owing to the inclusion of 35 individuals who were missing data for either cognitive ability or negative symptoms of motivation and pleasure and thus excluded from the 5-factor model.

**Figure 3.**
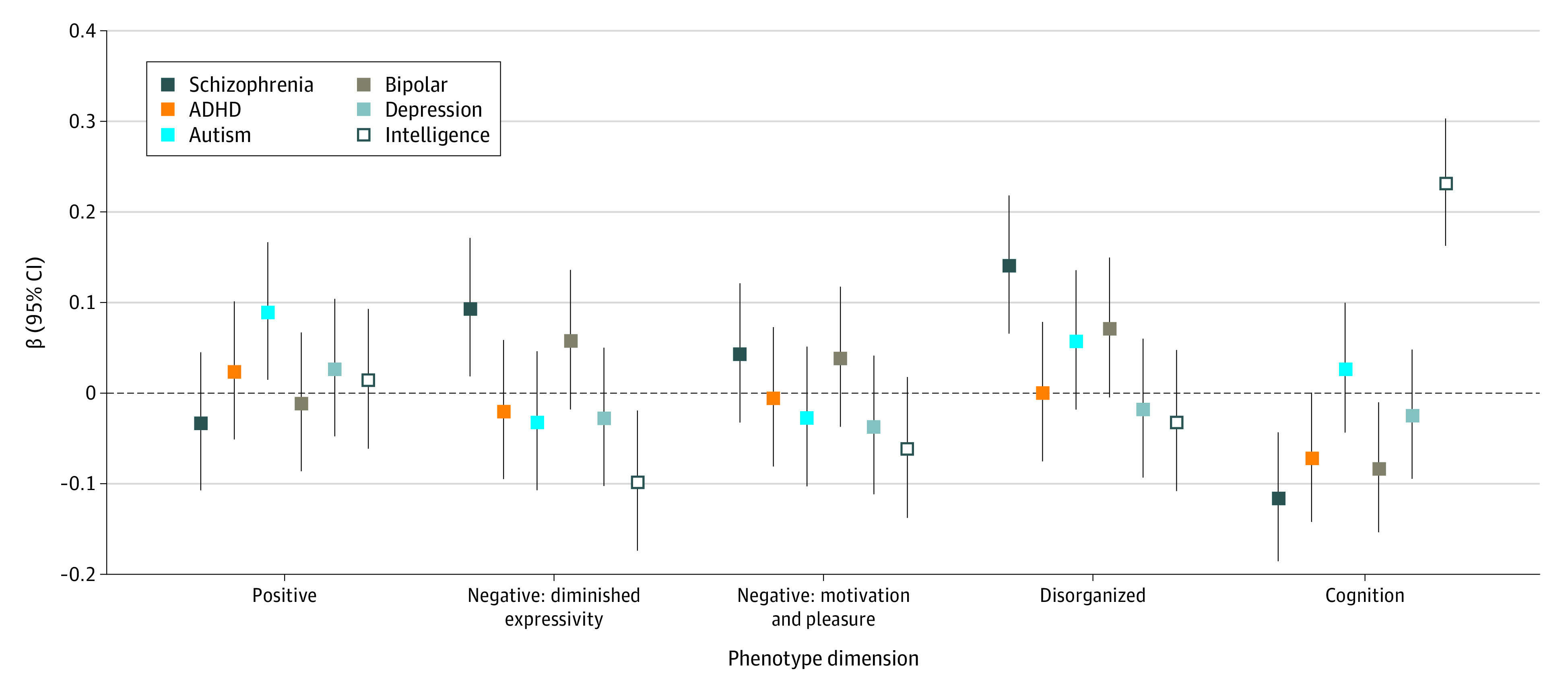
Association of Polygenic Risk Scores and 5-Factor Phenotype Dimensions Association of polygenic risk scores (PRSs) for schizophrenia, attention-deficit/hyperactivity disorder (ADHD), autism spectrum disorder, bipolar disorder, depression, and intelligence with phenotype dimensions derived from the 5-factor model in CardiffCOGS. Error bars represent the 95% CI of the β value. Dotted line represents a null model (values >0 indicate increased risk and values <0 indicate reduced risk).

Figure 3 and eTable 7 in the Supplement detail the associations between the 5-factor phenotype dimensions in CardiffCOGS and PRSs for bipolar disorder, depression, autism, ADHD, and intelligence at the threshold *P* ≤ .05. The only association that survived a Bonferroni correction for the number of tests conducted (n = 25; *P* < .002) was between the intelligence PRS and current cognitive ability (β = 0.23; 95% CI, 0.16 to 0.30; *P* = 1.52 × 10^−10^).

In meta-analysis for the 3-factor model, none of the PRSs for bipolar disorder, depression, autism, ADHD, or intelligence were significantly associated with any of the symptom dimensions after correction for multiple testing (eTable 8 in the Supplement).

The intelligence PRS was strongly associated with premorbid IQ as measured by the National Adult Reading Test (β = 0.33; 95% CI, 0.26-0.41; *P* = 4.76 × 10^−17^), whereas the schizophrenia PRS was not (β = −0.04; 95% CI, −0.12 to 0.04; *P* = .323). Premorbid IQ and the current cognitive ability dimension had a Pearson correlation of 0.50. We added premorbid IQ as a covariate in the regressions of the current cognitive ability dimension, as defined in the 5-factor CFA model, on the PRSs for schizophrenia and intelligence—a method that has been demonstrated to be preferable to the creation of change scores.^43^ The association between current cognitive ability and schizophrenia PRS remained significant (β = −0.08; 95% CI, −0.14 to −0.02; *P* = 8.50 × 10^−3^), whereas the comparison with intelligence PRS did not (β = 0.06; 95% CI, −0.01-0.12; *P* = .08).

## Discussion

In this study, we found evidence that higher levels of disorganized symptoms and lower levels of current cognitive ability were significantly associated with schizophrenia PRS, suggesting that these phenotypes are markers of increased genetic liability to schizophrenia. We also found that current cognitive performance in schizophrenia reflects genetic liabilities to both schizophrenia and intelligence.

Our first notable finding relates to the association between schizophrenia PRS and disorganized dimension scores in individuals with schizophrenia based on lifetime worst ratings; we found a significant association in the 5-factor model derived in our largest sample and in a meta-analysis of a 3-factor model across 3 samples. This finding is consistent with a twin study that found that the disorganized symptom dimension was a marker of genetic loading for psychotic disorders.^2^ We noted little evidence that the disorganized dimension was significantly associated with genetic liability to other neuropsychiatric disorders or intelligence, suggesting some specificity with schizophrenia genetic liability.

Previous studies assessing the relationships between schizophrenia PRS and symptom dimensions in schizophrenia have reported associations with a combined negative/disorganized dimension.^9,10^ Follow-up analyses in one of these studies of raw symptom scores found that this signal appeared to be influenced by the disorganized rather than the negative symptom scores.^10^ Our study clarifies the associations between schizophrenia PRS and symptom dimensions by including distinct disorganized and negative dimensions in the primary analysis. We found that, although schizophrenia PRS was nominally associated with the dimension relating to negative symptoms of diminished expressivity in our largest sample, this association attenuated when included in a model with the disorganized dimension and was not associated in the meta-analysis of the 3-factor model or with the raw phenotypes. Thus, our findings suggest that the association with genetic liability to schizophrenia is principally associated with disorganized rather than negative symptoms. These findings support the importance of developing better assessments and capturing subjective and observed data for disorganized symptoms in schizophrenia research, which may have particular relevance for clinical studies into basic symptoms^44^ and genetic high risk.

The lack of association between genetic liability for schizophrenia and positive symptoms is consistent with previous studies in individuals with schizophrenia.^2,9,10,13^ Our study extends these findings to show that, in people with schizophrenia, there is no association between positive symptoms and genetic liability for bipolar disorder, depression, ADHD, autism, or intelligence.

The second key finding from this study concerns the associations between schizophrenia PRS, intelligence PRS, and current cognitive ability. Although both sets of PRSs were associated with current cognitive ability, the intelligence PRS, but not the schizophrenia PRS, was associated with premorbid IQ as estimated by the National Adult Reading Test. Moreover, when adding premorbid IQ as a covariate, the association between current cognitive ability and schizophrenia PRS remained significant, but the association with intelligence PRS did not. These results suggest that current cognitive ability in individuals with schizophrenia is partly a function of premorbid IQ, influenced by genetic variants that contribute to variation in intelligence in the general population and additionally influenced by schizophrenia risk alleles. The latter could affect cognitive ability via processes intrinsic to schizophrenia pathophysiologic factors and/or via consequences of having schizophrenia, such as medication effects or social isolation. It is of interest that a recent study of 22q11.2 deletion syndrome also found evidence of an association between decline in intelligence and schizophrenia PRS.^45^ Another possibility is that schizophrenia PRS predominantly influences fluid intelligence (primary component of the MATRICS Consensus Cognitive Battery) as opposed to crystallized intelligence (primary component of the National Adult Reading Test), which may be more related to intelligence PRS.^46^ The genetic basis of cognitive impairment in schizophrenia is an important area for future research given the association between cognitive impairment and poor functional outcome in schizophrenia.^47^ Studies investigating the association between schizophrenia PRS and cognitive ability in patients with schizophrenia have reported inconsistent findings,^4,17^ which may be owing to differences in the duration between schizophrenia onset and cognitive assessment and the aspects of cognition measured.^48^

The phenotypic correlation between the disorganized and cognitive ability dimensions in our study was low (−0.12), consistent with previous studies,^49^ and in the model including all dimensions, schizophrenia PRS was significantly associated with both the disorganized and cognitive ability dimensions. These findings suggest that disorganized symptoms and current cognitive ability in patients with schizophrenia are independent markers of genetic liability to schizophrenia. In addition to disorganized symptoms and cognitive ability being influenced by common schizophrenia genetic risk factors, these and the other phenotypes assessed might be influenced by rarer schizophrenia genetic risk factors, genetic factors independent of schizophrenia liability, and a range of environmental factors.^1^

### Strengths and Limitations

A key strength of this study is the use of 3 independent samples, each with well-characterized symptom data derived from both interview and clinical case records focusing on lifetime symptoms. The symptom dimensions were identified from the Scale for the Assessment of Positive Symptoms and Scale for the Assessment of Negative Symptoms in all 3 samples, thus allowing for relatively high consistency of symptom dimension measures. The study included cognitive ability as well as symptom dimensions as distinct phenotypes and optimized phenotypic structure using confirmatory factor analysis.

However, the study findings should be interpreted in the context of several limitations. The results presented in this study are limited by the fact that cognitive data were not available in 2 of the samples, and thus these results require replication in other samples that have dimensional phenotypes including disorganized symptoms and cognitive measures. The lifetime symptom scores were based in part on retrospective reports during interviews, but their use in conjunction with contemporaneous clinical records will have increased the accuracy of the phenotypic measures. In addition, our analyses consisted of individuals of White European ancestry because the sample contained too few participants of non-European ancestry to analyze thoroughly. Thus, further studies are required to establish the generalizability of these findings to all people with schizophrenia.

## Conclusions

The results of this study suggest that variation in disorganized symptoms and cognitive ability in schizophrenia are independent markers of the extent to which individuals carry common genetic risk variants for schizophrenia. Moreover, cognitive performance in schizophrenia reflects genetic liabilities to both schizophrenia and intelligence. Further investigation of the genetic basis of these phenotypes and the underlying mechanisms may have potential to improve diagnosis, prognosis, and development of treatments for aspects of schizophrenia currently associated with poor outcomes.

## Notes

Supplement. **eAppendix 1.** Quality Control and Imputation of Genetic Data **eAppendix 2.** Goodness of Fit Indices for CFA **eFigure 1.** 3-Factor CFA Models **eFigure 2.** Schizophrenia PRS and Raw Summed Phenotypes **eTable 1.** Demographic Characteristics for Each Sample **eTable 2.** CFA Models in CardiffCOGS **eTable 3.** Schizophrenia PRS and 5-Factor Model for All *P* Value Thresholds **eTable 4.** Schizophrenia PRS Predicted by All CFA Dimensions **eTable 5.** Schizophrenia PRS and Raw Phenotype Scores **eTable 6.** Schizophrenia PRS Covarying for Potential Confounders **eTable 7.** Association of Related PRS With 5-Factor Model **eTable 8.** Related PRS Meta-analysis for 3-Factor Model eReferences

## References

[1] Rijsdijk FV, Gottesman II, McGuffin P, Cardno AG. Heritability estimates for psychotic symptom dimensions in twins with psychotic disorders. Am J Med Genet B Neuropsychiatr Genet. 2011;156B(1):89-98. doi:10.1002/ajmg.b.31145 21184588

[2] Cardno AG, Sham PC, Murray RM, McGuffin P. Twin study of symptom dimensions in psychoses. Br J Psychiatry. 2001;179:39-45. doi:10.1192/bjp.179.1.39 11435267

[3] Santoro ML, Ota V, de Jong S, . Polygenic risk score analyses of symptoms and treatment response in an antipsychotic-naive first episode of psychosis cohort. Transl Psychiatry. 2018;8(1):174. doi:10.1038/s41398-018-0230-7 30171181 PMC6119191

[4] Jonas KG, Lencz T, Li K, . Schizophrenia polygenic risk score and 20-year course of illness in psychotic disorders. Transl Psychiatry. 2019;9(1):300. doi:10.1038/s41398-019-0612-5 31727878 PMC6856168

[5] Cardno AG, Jones LA, Murphy KC, . Dimensions of psychosis in affected sibling pairs. Schizophr Bull. 1999;25(4):841-850. doi:10.1093/oxfordjournals.schbul.a033423 10667752

[6] Rietkerk T, Boks MP, Sommer IE, Liddle PF, Ophoff RA, Kahn RS. The genetics of symptom dimensions of schizophrenia: review and meta-analysis. Schizophr Res. 2008;102(1-3):197-205. doi:10.1016/j.schres.2008.01.023 18328672

[7] McGrath JA, Avramopoulos D, Lasseter VK, . Familiality of novel factorial dimensions of schizophrenia. Arch Gen Psychiatry. 2009;66(6):591-600. doi:10.1001/archgenpsychiatry.2009.56 19487624

[8] Cardno AG, Thomas K, McGuffin P. Clinical variables and genetic loading for schizophrenia: analysis of published Danish adoption study data. Schizophr Bull. 2002;28(3):393-399. doi:10.1093/oxfordjournals.schbul.a006948 12645672

[9] Ruderfer DM, Ripke S, McQuillin A, ; Bipolar Disorder and Schizophrenia Working Group of the Psychiatric Genomics Consortium. Genomic dissection of bipolar disorder and schizophrenia, including 28 subphenotypes. Cell. 2018;173(7):1705-1715.e16. doi:10.1016/j.cell.2018.05.046 29906448 PMC6432650

[10] Fanous AH, Zhou B, Aggen SH, ; Schizophrenia Psychiatric Genome-Wide Association Study (GWAS) Consortium. Genome-wide association study of clinical dimensions of schizophrenia: polygenic effect on disorganized symptoms. Am J Psychiatry. 2012;169(12):1309-1317. doi:10.1176/appi.ajp.2012.12020218 23212062 PMC3646712

[11] Jones HJ, Stergiakouli E, Tansey KE, . Phenotypic manifestation of genetic risk for schizophrenia during adolescence in the general population. JAMA Psychiatry. 2016;73(3):221-228. doi:10.1001/jamapsychiatry.2015.3058 26818099 PMC5024747

[12] Pain O, Dudbridge F, Cardno AG, . Genome-wide analysis of adolescent psychotic-like experiences shows genetic overlap with psychiatric disorders. Am J Med Genet B Neuropsychiatr Genet. 2018;177(4):416-425. doi:10.1002/ajmg.b.32630 29603866 PMC6001485

[13] Bigdeli TB, Peterson RE, Ripke S, ; Genome-wide Association Study of Clinical Features in the Schizophrenia Psychiatric Genomics Consortium. Confirmation of polygenic effect on negative symptoms. bioRxiv. 2017:161349. doi:10.1101/161349

[14] Strauss GP, Nuñez A, Ahmed AO, . The latent structure of negative symptoms in schizophrenia. JAMA Psychiatry. 2018;75(12):1271-1279. doi:10.1001/jamapsychiatry.2018.2475 30208377 PMC6583036

[15] Strauss GP, Horan WP, Kirkpatrick B, . Deconstructing negative symptoms of schizophrenia: avolition-apathy and diminished expression clusters predict clinical presentation and functional outcome. J Psychiatr Res. 2013;47(6):783-790. doi:10.1016/j.jpsychires.2013.01.015 23453820 PMC3686506

[16] Savage JE, Jansen PR, Stringer S, . Genome-wide association meta-analysis in 269,867 individuals identifies new genetic and functional links to intelligence. Nat Genet. 2018;50(7):912-919. doi:10.1038/s41588-018-0152-6 29942086 PMC6411041

[17] Richards AL, Pardiñas AF, Frizzati A, ; GROUP Investigators; EUGEI WP2 Group; Schizophrenia Working Group of the Psychiatric Genomics Consortium. The relationship between polygenic risk scores and cognition in schizophrenia. Schizophr Bull. 2020;46(2):336-344. doi:10.1093/schbul/sbz061 31206164 PMC7442352

[18] Dickinson D, Zaidman SR, Giangrande EJ, Eisenberg DP, Gregory MD, Berman KF. Distinct polygenic score profiles in schizophrenia subgroups with different trajectories of cognitive development. Am J Psychiatry. 2020;177(4):298-307. doi:10.1176/appi.ajp.2019.19050527 31838871 PMC9627722

[19] van Scheltinga AF, Bakker SC, van Haren NE, ; Psychiatric Genome-Wide Association Study (GWAS) Consortium. Schizophrenia genetic variants are not associated with intelligence. Psychol Med. 2013;43(12):2563-2570. doi:10.1017/S0033291713000196 23410598 PMC4743754

[20] Legge SE, Dennison CA, Pardiñas AF, . Clinical indicators of treatment-resistant psychosis. Br J Psychiatry. 2020;216(5):259-266. doi:10.1192/bjp.2019.120 31155017

[21] Williams NM, Green EK, Macgregor S, . Variation at the DAOA/G30 locus influences susceptibility to major mood episodes but not psychosis in schizophrenia and bipolar disorder. Arch Gen Psychiatry. 2006;63(4):366-373. doi:10.1001/archpsyc.63.4.366 16585465

[22] Wing JK, Babor T, Brugha T, ; Schedules for Clinical Assessment in Neuropsychiatry. SCAN: Schedules for Clinical Assessment in Neuropsychiatry. Arch Gen Psychiatry. 1990;47(6):589-593. doi:10.1001/archpsyc.1990.01810180089012 2190539

[23] American Psychiatric Association. Diagnostic and Statistical Manual of Mental Disorders: DSM-IV. 4th ed. American Psychiatric Association; 1994.

[24] World Health Organization. The ICD-10 Classification of Mental and Behavioural Disorders: Clinical Descriptions and Diagnostic Guidelines. World Health Organization; 1992.

[25] Andreasen NC. Scale for the Assessment of Positive Symptoms. University of Iowa Press; 1984.

[26] Andreasen NC. The Scale for the Assessment of Negative Symptoms (SANS): conceptual and theoretical foundations. Br J Psychiatry Suppl. 1989;(7):49-58. doi:10.1192/S0007125000291496 2695141

[27] Nuechterlein KH, Green MF, Kern RS, . The MATRICS Consensus Cognitive Battery, part 1: test selection, reliability, and validity. Am J Psychiatry. 2008;165(2):203-213. doi:10.1176/appi.ajp.2007.07010042 18172019

[28] Nelson HE. The National Adult Reading Test (NART). NFER-Nelcon; 1991.

[29] Lynham AJ, Hubbard L, Tansey KE, . Examining cognition across the bipolar/schizophrenia diagnostic spectrum. J Psychiatry Neurosci. 2018;43(4):245-253. doi:10.1503/jpn.170076 29947606 PMC6019354

[30] Chang CC, Chow CC, Tellier LC, Vattikuti S, Purcell SM, Lee JJ. Second-generation PLINK: rising to the challenge of larger and richer datasets. Gigascience. 2015;4:7. doi:10.1186/s13742-015-0047-8 25722852 PMC4342193

[31] Schizophrenia Working Group of the Psychiatric Genomics Consortium. Biological insights from 108 schizophrenia-associated genetic loci. Nature. 2014;511(7510):421-427. doi:10.1038/nature13595 25056061 PMC4112379

[32] Euesden J, Lewis CM, O’Reilly PF. PRSice: polygenic risk score software. Bioinformatics. 2015;31(9):1466-1468. doi:10.1093/bioinformatics/btu848 25550326 PMC4410663

[33] Wray NR, Lee SH, Mehta D, Vinkhuyzen AA, Dudbridge F, Middeldorp CM. Research review: polygenic methods and their application to psychiatric traits. J Child Psychol Psychiatry. 2014;55(10):1068-1087. doi:10.1111/jcpp.12295 25132410

[34] Ripke S, Walters JT, O’Donovan MC; The Schizophrenia Working Group of the Psychiatric Genomics Consortium. Mapping genomic loci prioritises genes and implicates synaptic biology in schizophrenia. medRxiv. 2020:2020.2009.2012.20192922. doi:10.1101/2020.09.12.20192922

[35] Stahl EA, Breen G, Forstner AJ, ; eQTLGen Consortium; BIOS Consortium; Bipolar Disorder Working Group of the Psychiatric Genomics Consortium. Genome-wide association study identifies 30 loci associated with bipolar disorder. Nat Genet. 2019;51(5):793-803. doi:10.1038/s41588-019-0397-8 31043756 PMC6956732

[36] Howard DM, Adams MJ, Clarke TK, ; 23andMe Research Team; Major Depressive Disorder Working Group of the Psychiatric Genomics Consortium. Genome-wide meta-analysis of depression identifies 102 independent variants and highlights the importance of the prefrontal brain regions. Nat Neurosci. 2019;22(3):343-352. doi:10.1038/s41593-018-0326-7 30718901 PMC6522363

[37] Grove J, Ripke S, Als TD, ; Autism Spectrum Disorder Working Group of the Psychiatric Genomics Consortium; BUPGEN; Major Depressive Disorder Working Group of the Psychiatric Genomics Consortium; 23andMe Research Team. Identification of common genetic risk variants for autism spectrum disorder. Nat Genet. 2019;51(3):431-444. doi:10.1038/s41588-019-0344-8 30804558 PMC6454898

[38] Demontis D, Walters RK, Martin J, ; ADHD Working Group of the Psychiatric Genomics Consortium (PGC); Early Lifecourse & Genetic Epidemiology (EAGLE) Consortium; 23andMe Research Team. Discovery of the first genome-wide significant risk loci for attention deficit/hyperactivity disorder. Nat Genet. 2019;51(1):63-75. doi:10.1038/s41588-018-0269-7 30478444 PMC6481311

[39] Rosseel Y. lavaan: an R package for structural equation modeling. J Statistical Software. 2012;48(2):1-36. doi:10.18637/jss.v048.i02

[40] Schreiber JB, Nora A, Stage FK, Barlow EA, King J. Reporting structural equation modeling and confirmatory factor analysis results: a review. J Educational Res. 2006;99(6):323-337. doi:10.3200/JOER.99.6.323-338

[41] Hu LT, Bentler PM. Cutoff criteria for fit indexes in covariance structure analysis: conventional criteria versus new alternatives. Structural Equation Modeling. 1999;6(1):1-55. doi:10.1080/10705519909540118

[42] Schwarzer G. meta: an R package for meta-analysis. R News. 2007;7(3):40-45.

[43] Griffin D, Murray S, Gonzalez R. Difference score correlations in relationship research: a conceptual primer. Personal Relationships. 1999;6(4):505-518. doi:10.1111/j.1475-6811.1999.tb00206.x

[44] Schultze-Lutter F. Subjective symptoms of schizophrenia in research and the clinic: the basic symptom concept. Schizophr Bull. 2009;35(1):5-8. doi:10.1093/schbul/sbn139 19074497 PMC2643966

[45] Davies RW, Fiksinski AM, Breetvelt EJ, ; International 22q11.2 Brain and Behavior Consortium. Using common genetic variation to examine phenotypic expression and risk prediction in 22q11.2 deletion syndrome. Nat Med. 2020;26(12):1912-1918. doi:10.1038/s41591-020-1103-1 33169016 PMC7975627

[46] Carey C, Huang Y, Strong R, . Shared and distinct genetic influences between cognitive domains and psychiatric disorder risk based on genome-wide data. bioRxiv. 2020:2020.2009.2016.297408.

[47] Mucci A, Galderisi S, Gibertoni D, ; Italian Network for Research on Psychoses. Factors associated with real-life functioning in persons with schizophrenia in a 4-year follow-up study of the Italian Network for Research on Psychoses. JAMA Psychiatry. 2021;78(5):550-559. doi:10.1001/jamapsychiatry.2020.4614 33566071 PMC7876615

[48] Shafee R, Nanda P, Padmanabhan JL, . Polygenic risk for schizophrenia and measured domains of cognition in individuals with psychosis and controls. Transl Psychiatry. 2018;8(1):78. doi:10.1038/s41398-018-0124-8 29643358 PMC5895806

[49] Dominguez MdeG, Viechtbauer W, Simons CJ, van Os J, Krabbendam L. Are psychotic psychopathology and neurocognition orthogonal? a systematic review of their associations. Psychol Bull. 2009;135(1):157-171. doi:10.1037/a0014415 19210058

