# Supplementary material for: Associations Between Schizophrenia Polygenic Liability, Symptom Dimensions, and Cognitive Ability in Schizophrenia
Source: JAMA Psychiatry. 2021 Aug 4;78(10):1143–51. doi: 10.1001/jamapsychiatry.2021.1961 (PMC8340009; doi:10.1001/jamapsychiatry.2021.1961)
Supplement: Supplement. — eAppendix 1. Quality Control and Imputation of Genetic Data eAppendix 2. Goodness of Fit Indices for CFA eFigure 1. 3-Factor CFA Models eFigure 2. Schizophrenia PRS and Raw Summed Phenotypes eTable 1. Demographic Characteristics for Each Sample eTable 2. CFA Models in CardiffCOGS eTable 3. Schizophrenia PRS and 5-Factor Model for All P Value Thresholds eTable 4. Schizophrenia PRS Predicted by All CFA Dimensions eTable 5. Schizophrenia PRS and Raw Phenotype Scores eTable 6. Schizophrenia PRS Covarying for Potential Confounders eTable 7. Association of Related PRS With 5-Factor Model eTable 8. Related PRS Meta-analysis for 3-Factor Model eReferences [file jamapsychiatry-e211961-s001.pdf]

Supplementary Online Content

Legge SE, Cardno AG, Allardyce J, et al. Associations between schizophrenia polygenic liability, symptom dimensions, and cognitive ability in schizophrenia. *JAMA Psychiatry*. Published online August 4, 2021.
doi:10.1001/jamapsychiatry.2021.1961

eAppendix 1. Quality Control and Imputation of Genetic Data

eAppendix 2. Goodness of Fit Indices for CFA

eFigure 1. 3-Factor CFA Models

eFigure 2. Schizophrenia PRS and Raw Summed Phenotypes

eTable 1. Demographic Characteristics for Each Sample

eTable 2. CFA Models in CardiffCOGS

eTable 3. Schizophrenia PRS and 5-Factor Model for All *P* Value Thresholds

eTable 4. Schizophrenia PRS Predicted by All CFA Dimensions

eTable 5. Schizophrenia PRS and Raw Phenotype Scores

eTable 6. Schizophrenia PRS Covarying for Potential Confounders

eTable 7. Association of Related PRS With 5-Factor Model

eTable 8. Related PRS Meta-analysis for 3-Factor Model

eReferences

This supplementary material has been provided by the authors to give readers additional information about their work.

eAppendix 1: Quality control and imputation of genetic data

All samples were genotyped on the Illumina HumanOmniExpress (version 8 or 12). The following quality control exclusions were applied to SNPs: minor allele frequency (MAF) < 0.01, genotyping rates < 0.95, and Hardy-Weinberg equilibrium (HWE) p-value < 1×10^{-6}. Individuals with sample missingness > 0.05 were excluded. GenomeHarmoniser[1] was used to align SNPs to the Haplotype Reference Consortium (HRC)[2] prior to imputation on the Michigan Imputation Server[3] using the HRC v1.1 as the reference panel. Samples on different versions of the genotyping platform were merged after this initial round of quality control, and the same measures were applied to the merged dataset. The following quality control exclusions were then applied to best estimate genotype data in addition to those listed above; imputation quality score (R^2) < 0.8 and HWE p-value < 1×10^{-4}.

Genetic principal components representing ancestry were derived using PLINK v2.0[4] using SNPs with low levels of linkage disequilibrium ($r^2 < 0.2$ and 500kb window; criteria used by the PGC[5]). All genetic analyses were restricted to those of European ancestry, as assessed by principal components. Our sample contained too few participants of non-European ancestry to analyse effectively. First-degree relatives ($\pi > 0.4$) both within and between samples were identified and one member of each related pair was removed, preferentially retaining samples that had more complete phenotype data and otherwise removed at random.

eAppendix 2: Goodness of fit indices for CFA

The standardized root mean square residual (SRMR) is an absolute fit index that compares the residuals of the hypothesised model covariance to the sample covariance matrix. The root mean square error of approximation (RMSEA) is a parsimony-adjusted index (favours the model with the lesser number of parameters) that assesses the extent to which the hypothesised model fits the sample covariance matrix and is robust to issues relating to sample size. The comparative fit index (CFI) is an incremental fit index that compares the hypothesised model with that of a null model[6]. Model fit was guided by standard interpretations[7,8] of the goodness of fit indices; CFI values of at least 0.95, RMSEA values lower than 0.06, and an SRMR value lower than 0.08.

eFigure 1: 3-factor CFA models

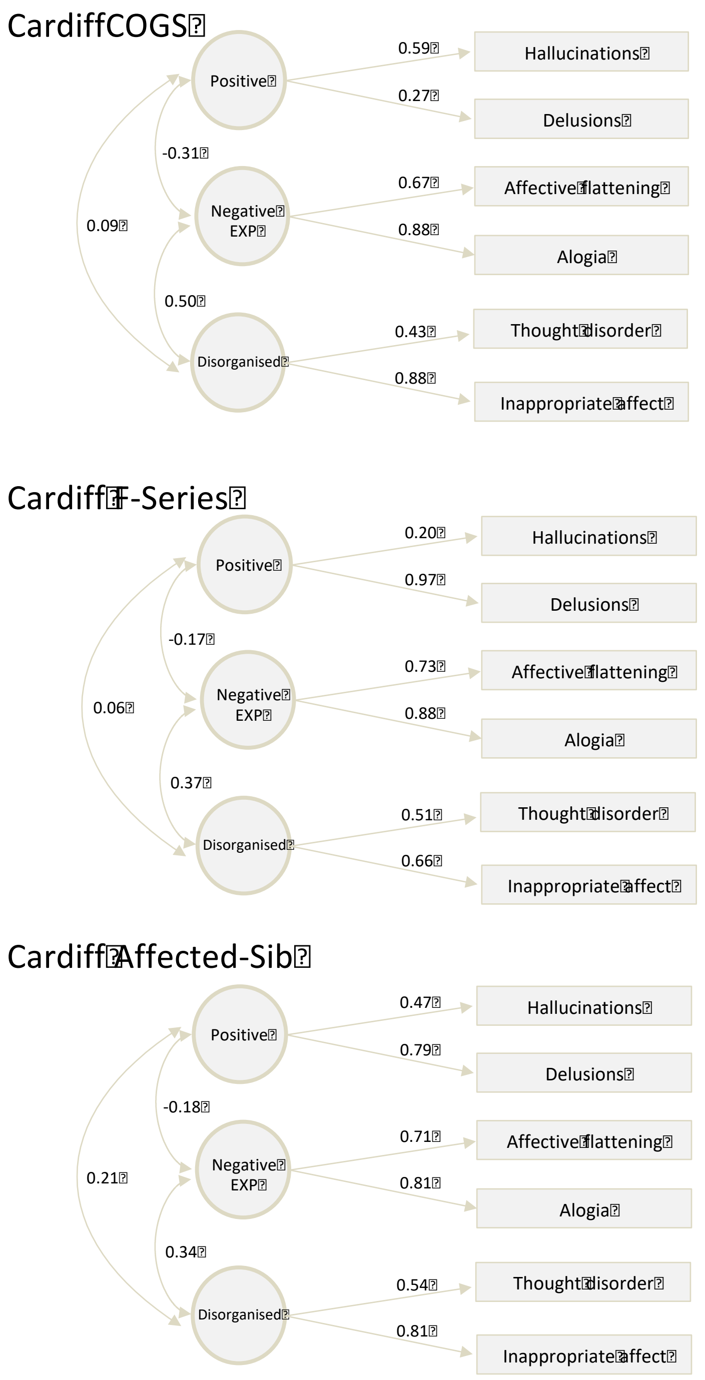

Structure of 3-factor phenotype domains derived by CFA in CardiffCOGS, Cardiff F-series and Cardiff Affected-Sib samples. Boxes on the right detail contributing phenotypes and circles represent latent factors created by CFA. Solid lines represent standardised factor loadings and curved lines represent the correlation among phenotype dimensions. The model was a good fit for the data in each sample; CardiffCOGS (CFI=0.99; RMSEA [95% CI]=0.05 [0.02,0.07]; SRMR=0.04), Cardiff F-series (CFI=1.00; RMSEA [95% CI]=0.00 [0.00,0.05]; SRMR=0.04), and Cardiff Affected-Sib (CFI=0.99; RMSEA [95% CI]=0.05 [0.00,0.09]; SRMR=0.04).

eFigure 2: Schizophrenia PRS and raw summed phenotypes

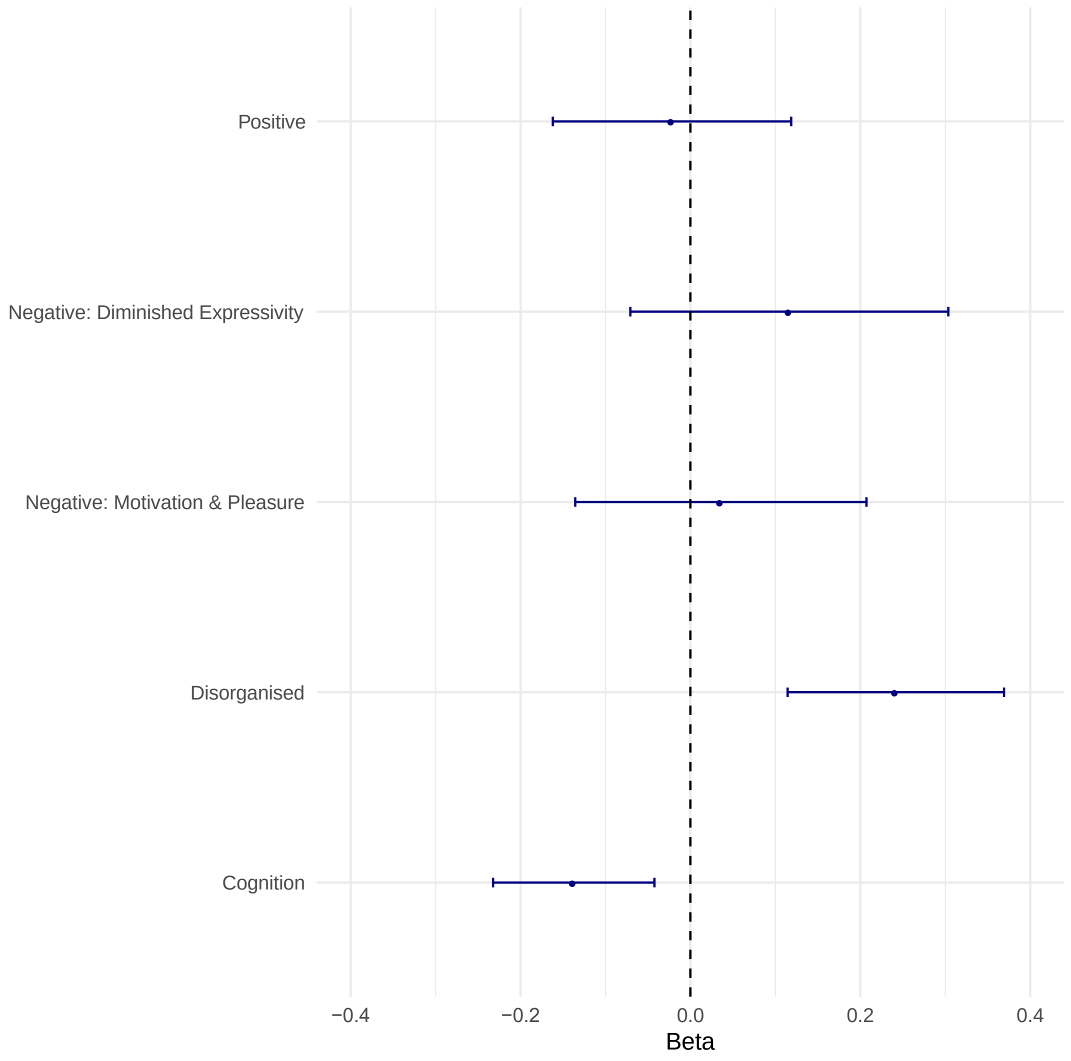

Association of polygenic risk scores (PRS) for schizophrenia and phenotype domains derived from summing the SAPS and SANS raw scores that are used to derive phenotype domains in the 5-factor model in CardiffCOGS. Error bars represent the 95% confidence interval of the beta value. Dotted line represents a null model (values > 0 indicate increased risk and values < 0 indicate reduced risk).

eTable 1: Demographic characteristics for each sample

	CardiffCOGS	Cardiff F-series	Cardiff Affected-Sib
Total number of participants	697	383	140
Male (% total)	462 (66.28%)	265 (69.19%)	90 (64.29%)
Mean age at interview [SD]	43.32 [11.95]	42.94 [14.06]	42.22 [12.97]
Mean age at onset of psychosis [SD]	24.75 [8.94]	23.92 [8.37]	23.88 [7.45]
Antipsychotic treatment resistance (%)	366 (55.45%)	68 (21.45%)	53 (39.55%)
Diagnosis			
Schizophrenia (% total)	580 (83.21%)	357 (93.21%)	138 (98.57%)
Schizoaffective depressed (% total)	117 (16.79%)	26 (6.79%)	2 (1.43%)

Details of demographic characteristics relating to each cross-sectional sample.

eTable 2: CFA models in CardiffCOGS

Phenotype	N (935)	Mean	SD	Min	Max	M1	M2	M3	M4	M5	M6	M7	M8
SAPS global hallucinations	909	2.76	1.57	0	5	1	1	1	1	1	1	1	1
SAPS global delusions	909	3.37	0.99	0	5	1	1	1	1	1	1	1	1
SANS global affective flattening	906	1.63	1.47	0	5	1	1	2	2	2	2	2	2
SANS global alogia	907	1.34	1.54	0	5	1	1	2	2	2	2	2	2
SANS global anhedonia/asociality	906	2.37	1.43	0	5	1	1	3	3	3	3	2	2
SANS global avolition/apathy	909	2.18	1.28	0	5	1	1	3	3	3	3	2	2
SAPS global positive formal thought disorder	907	1.08	1.39	0	5	1	1	4	4	4	4	3	2
SANS inappropriate affect	906	0.31	0.83	0	4	1	1	4	4	4	4	3	2
MATRICS problem solving (mazes)	888	-1.53	1.31	-6.2	1.17	1	2	5	5	5			
MATRICS verbal learning (HVLT)	888	-2.45	1.57	-6.63	1.59	1	2	5	5	5			
MATRICS visual learning (BVMT)	888	-1.64	1.26	-5.49	1.52	1	2	5	5	5			
MATRICS attention (CPT)	888	-1.48	1.23	-5.52	2.25	1	2	5	5	5			
MATRICS speed of processing	887	-1.94	1.14	-6.13	1.54	1	2	5	5	5			
MATRICS working memory	886	-1.66	1.24	-5.29	1.91	1	2	5	5	5			
MATRICS social cognition (MSCEIT)	887	-1.1	1.2	-5.01	3.04				5				
SAPS global bizarre behaviour	909	2.24	1.24	0	5					4			
**CFI**						0.76	0.956	0.985	0.985	0.978	0.984	0.948	0.937
**RMSEA**						0.123	0.058	0.036	0.035	0.041	0.048	0.079	0.075
**RMSEA L95**						0.115	0.051	0.028	0.028	0.034	0.032	0.065	0.059
**RMSEA U95**						0.131	0.065	0.045	0.043	0.048	0.065	0.093	0.091
**SRMR**						0.127	0.066	0.045	0.046	0.051	0.043	0.063	0.073

Phenotypes used to identify CFA models in CardiffCOGS. Columns represent the phenotype entered into the CFA models (Phenotype), and the numbers of individuals with complete data (N), the mean value (Mean), standard deviation (SD), minimum value (Min) and maximum value (Max) for each phenotype. Columns labelled M1-M8 represent the eight combinations of phenotypes entered into a CFA model. Rows labelled CFI, RMSEA, RMSEA L95, RMSEA U95, and SRMR represent the fit statistics for M1-M8. Model 3 is highlighted in red and was selected as the best fitting model based on fit statistics but also based on theoretical knowledge.

eTable 3: Schizophrenia PRS and 5-factor model for all p-value thresholds

Dimension: Positive						
**Pthres**	**beta**	**l95**	**u95**	**se**	**r2**	**p**
5.00E-08	0.003	-0.073	0.079	0.0387	-0.0015	0.936
1.00E-07	-0.015	-0.090	0.061	0.0386	-0.0013	0.706
1.00E-06	-0.008	-0.084	0.068	0.0387	-0.0014	0.841
1.00E-05	-0.012	-0.088	0.064	0.0388	-0.0014	0.763
0.0001	-0.021	-0.097	0.055	0.0387	-0.0011	0.592
0.001	-0.028	-0.104	0.048	0.0388	-0.0007	0.474
0.05	-0.031	-0.107	0.045	0.0388	-0.0005	0.422
0.1	-0.030	-0.107	0.046	0.0388	-0.0006	0.434
0.2	-0.021	-0.097	0.055	0.0388	-0.0011	0.586
0.5	-0.022	-0.099	0.054	0.0389	-0.0010	0.564
Dimension: Negative symptoms of diminished expressivity						
**Pthres**	**beta**	**l95**	**u95**	**se**	**r2**	**p**
5.00E-08	0.034	-0.043	0.110	0.0389	-0.0004	0.387
1.00E-07	0.029	-0.047	0.105	0.0389	-0.0007	0.457
1.00E-06	0.018	-0.059	0.094	0.0390	-0.0012	0.645
1.00E-05	0.013	-0.064	0.090	0.0390	-0.0014	0.738
0.0001	-0.022	-0.098	0.055	0.0390	-0.0010	0.575
0.001	0.045	-0.032	0.121	0.0390	0.0005	0.254
0.05	0.095	0.018	0.171	0.0389	0.0075	0.015
0.1	0.095	0.018	0.171	0.0389	0.0074	0.015
0.2	0.087	0.010	0.163	0.0389	0.0060	0.026
0.5	0.085	0.009	0.162	0.0390	0.0058	0.029
1	0.089	0.012	0.165	0.0390	0.0064	0.023
Dimension: Negative symptoms of motivation and pleasure						
**Pthres**	**beta**	**l95**	**u95**	**se**	**r2**	**p**
5.00E-08	0.030	-0.047	0.106	0.0390	-0.0006	0.446
1.00E-07	0.007	-0.070	0.083	0.0390	-0.0015	0.865
1.00E-06	0.008	-0.068	0.085	0.0391	-0.0015	0.830
1.00E-05	-0.002	-0.079	0.074	0.0392	-0.0015	0.951
0.0001	-0.041	-0.118	0.035	0.0390	0.0002	0.289
0.001	0.018	-0.059	0.095	0.0392	-0.0012	0.649
0.05	0.044	-0.033	0.121	0.0391	0.0004	0.258
0.1	0.048	-0.029	0.124	0.0392	0.0007	0.224
0.2	0.049	-0.028	0.126	0.0392	0.0009	0.212
0.5	0.056	-0.021	0.133	0.0392	0.0016	0.154
1	0.057	-0.020	0.134	0.0391	0.0017	0.144
Dimension: Disorganised symptoms						
**Pthres**	**beta**	**l95**	**u95**	**se**	**r2**	**p**
5.00E-08	0.078	0.002	0.155	0.0389	0.0047	0.045
1.00E-07	0.062	-0.015	0.138	0.0389	0.0023	0.114
1.00E-06	0.064	-0.013	0.140	0.0390	0.0025	0.103
1.00E-05	0.061	-0.016	0.138	0.0391	0.0022	0.121
0.0001	0.027	-0.050	0.104	0.0391	-0.0008	0.492
0.001	0.083	0.007	0.160	0.0391	0.0054	0.033
0.05	0.142	0.066	0.218	0.0388	0.0186	2.80x10-4
0.1	0.136	0.060	0.212	0.0389	0.0170	4.98x10-4
0.2	0.119	0.042	0.195	0.0390	0.0126	2.36x10-3
0.5	0.109	0.032	0.186	0.0390	0.0104	5.33x10-3
1	0.109	0.033	0.186	0.0390	0.0104	5.22x10-3

Dimension: Current cognitive ability						
**Pthres**	**beta**	**l95**	**u95**	**se**	**r2**	**p**
5.00E-08	-0.041	-0.113	0.030	0.0363	0.0004	0.254
1.00E-07	-0.042	-0.113	0.030	0.0363	0.0004	0.251
1.00E-06	-0.037	-0.109	0.034	0.0363	0.0001	0.306
1.00E-05	-0.037	-0.108	0.035	0.0364	0.0000	0.314
0.0001	-0.031	-0.103	0.040	0.0364	-0.0003	0.388
0.001	-0.041	-0.113	0.030	0.0365	0.0004	0.259
0.05	-0.115	-0.186	-0.043	0.0362	0.0118	0.002
0.1	-0.111	-0.183	-0.040	0.0362	0.0111	0.002
0.2	-0.108	-0.179	-0.037	0.0362	0.0103	0.003
0.5	-0.096	-0.167	-0.025	0.0363	0.0079	0.009
1	-0.098	-0.169	-0.027	0.0363	0.0083	0.007

Association of schizophrenia PRS and 5-factor model dimension scores in CardiffCOGS. Columns represent the p-value threshold used in the training GWAS dataset (Pthres), beta association value, lower 95% confidence interval of the beta (l95), upper 95% confidence interval of the beta (u95), standard error (se), variance explained (r2) and association p-value (p).

eTable 4: Schizophrenia PRS predicted by all CFA dimensions

**Phenotype dimension**	**β (95% CI)**	**SE**	**P**
Positive	-0.104 (-0.222, 0.014)	0.060	0.083
Negative: Diminished expressivity	0.185 (-0.440, 0.070)	0.130	0.154
Negative: Motivation & pleasure	0.036 (-0.137, 0.209)	0.088	0.682
Disorganised	0.226 (0.095, 0.358)	0.067	7.51×10^{-4}
Cognition	-0.196 (-0.318, -0.075)	0.062	1.61×10^{-3}

Association of schizophrenia PRS and all phenotype dimensions included simultaneously. Columns represent the phenotype, beta association value (β), and 95% confidence intervals of the beta (95% CI), standard error (se), and association p-value (p).

eTable 5: Schizophrenia PRS and raw phenotype scores

Phenotype dimension	β (95% CI)	SE	P
Positive	-0.022 (-0.162, 0.119)	0.071	0.761
Negative: Diminished expressivity	0.116 (-0.071, 0.304)	0.095	0.223
Negative: Motivation & pleasure	0.036 (-0.136, 0.207)	0.087	0.682
Disorganised	0.242 (0.114, 0.369)	0.065	2.12×10^{-4}
Cognition	-0.137 (-0.233, -0.042)	0.048	4.63×10^{-3}

Association of schizophrenia PRS and raw phenotype symptom scores. Columns represent the phenotype, beta association value (β), and 95% confidence intervals of the beta (95% CI), standard error (se), and association p-value (p).

eTable 6: Schizophrenia PRS covarying for potential confounders

	CardiffCOGS (n=662)			
**Phenotype dimension**	**β (95% CI)**	**SE**	**R^2**	**P**
Positive	-0.06 (-0.14, 0.02)	0.040	0.002	0.112
Negative: Diminished expressivity	0.09 (0.02, 0.17)	0.040	0.008	0.016
Negative: Motivation & pleasure	0.03 (-0.05, 0.11)	0.040	<0.001	0.431
Disorganised	0.15 (0.07, 0.23)	0.040	0.021	2.27×10^{-4}
Cognition	-0.09 (-0.16, -0.01)	0.037	0.006	0.020

Association of schizophrenia PRS and phenotype dimensions from 5-factor model in CardiffCOGS covarying for age of onset of psychosis and treatment resistance. Rows refer to each phenotype dimension as defined by CFA analyses. Columns refer to the beta and 95% confidence interval (β (95% CI)), standard error (SE), observed variance explained (R^2) by schizophrenia PRS, and p-value (P) from regression analyses.

eTable 7: Association of related PRS with 5-factor model

Dimension: Positive						
**PRS**	**beta**	**l95**	**u95**	**se**	**r2**	**p**
Bipolar	-0.010	-0.086	0.067	0.039	-0.001	0.803
Depression	0.028	-0.048	0.104	0.039	-0.001	0.467
Autism	0.091	0.015	0.166	0.039	0.007	0.020
ADHD	0.025	-0.051	0.101	0.039	-0.001	0.520
Intelligence	0.016	-0.061	0.093	0.039	-0.001	0.690
Dimension: Negative symptoms of diminished expressivity						
**PRS**	**beta**	**l95**	**u95**	**se**	**r2**	**p**
Bipolar	0.059	-0.018	0.136	0.039	0.002	0.133
Depression	-0.026	-0.103	0.050	0.039	-0.001	0.500
Autism	-0.031	-0.107	0.046	0.039	-0.001	0.433
ADHD	-0.018	-0.095	0.058	0.039	-0.001	0.641
Intelligence	-0.097	-0.174	-0.019	0.039	0.008	0.014
Dimension: Negative symptoms of motivation and pleasure						
**PRS**	**beta**	**l95**	**u95**	**se**	**r2**	**p**
Bipolar	0.040	-0.037	0.117	0.039	0.000	0.309
Depression	-0.035	-0.112	0.041	0.039	0.000	0.366
Autism	-0.025	-0.102	0.052	0.039	-0.001	0.517
ADHD	-0.004	-0.081	0.073	0.039	-0.002	0.914
Intelligence	-0.060	-0.138	0.018	0.040	0.002	0.129
Dimension: Disorganised symptoms						
**PRS**	**beta**	**l95**	**u95**	**se**	**r2**	**p**
Bipolar	0.072	-0.005	0.150	0.039	0.004	0.066
Depression	-0.017	-0.093	0.060	0.039	-0.001	0.670
Autism	0.059	-0.018	0.136	0.039	0.002	0.134
ADHD	0.002	-0.075	0.079	0.039	-0.002	0.968
Intelligence	-0.030	-0.108	0.048	0.040	-0.001	0.445
Dimension: Current cognitive ability						
**PRS**	**beta**	**l95**	**u95**	**se**	**r2**	**p**
Bipolar	-0.082	-0.154	-0.010	0.037	0.005	0.025
Depression	-0.023	-0.095	0.048	0.036	-0.001	0.521
Autism	0.028	-0.044	0.100	0.036	-0.001	0.444
ADHD	-0.071	-0.142	0.000	0.036	0.004	0.052
Intelligence	0.233	0.163	0.303	0.036	0.052	1.52E-10

Association of related PRS and 5-factor dimension scores in CardiffCOGS. Columns represent the PRS tested, beta association value, lower 95% confidence interval of the beta (l95), upper 95% confidence interval of the beta (u95), standard error (se), variance explained (r2) and association p-value (p).

eTable 8: Related PRS meta-analysis for 3-factor model

Dimension: Positive symptoms									
**PRS**	**Study**	**N**	**Beta**	**L95**	**U95**	**SE**	**P-val**	**Q**	**Q p-val**
Bipolar	CardiffCOGS	697	-0.026	-0.119	0.067	0.047	0.588		
Bipolar	Cardiff F-series	383	-0.027	-0.073	0.020	0.024	0.259		
Bipolar	Cardiff Affected-Sib	140	-0.193	-0.381	-0.006	0.094	0.043		
**Bipolar**	**Meta-analysis**	**1220**	**-0.035**	**-0.075**	**0.006**	**0.021**	**0.095**	**2.971**	**0.226**
Depression	CardiffCOGS	697	0.031	-0.059	0.121	0.046	0.500		
Depression	Cardiff F-series	383	-0.015	-0.059	0.029	0.022	0.504		
Depression	Cardiff Affected-Sib	140	0.029	-0.167	0.225	0.099	0.767		
**Depression**	**Meta-analysis**	**1220**	**-0.005**	**-0.043**	**0.034**	**0.020**	**0.810**	**0.934**	**0.627**
Autism	CardiffCOGS	697	0.132	0.041	0.223	0.046	0.005		
Autism	Cardiff F-series	383	0.037	-0.008	0.082	0.023	0.109		
Autism	Cardiff Affected-Sib	140	-0.055	-0.255	0.145	0.101	0.585		
**Autism**	**Meta-analysis**	**1220**	**0.051**	**0.012**	**0.091**	**0.020**	**0.011**	**4.531**	**0.104**
ADHD	CardiffCOGS	697	0.037	-0.052	0.126	0.045	0.412		
ADHD	Cardiff F-series	383	0.021	-0.025	0.066	0.023	0.367		
ADHD	Cardiff Affected-Sib	140	-0.170	-0.381	0.042	0.107	0.115		
**ADHD**	**Meta-analysis**	**1220**	**0.017**	**-0.022**	**0.057**	**0.020**	**0.393**	**3.289**	**0.193**
Intelligence	CardiffCOGS	697	0.062	-0.030	0.155	0.047	0.187		
Intelligence	Cardiff F-series	383	0.011	-0.036	0.058	0.024	0.645		
Intelligence	Cardiff Affected-Sib	140	-0.011	-0.209	0.186	0.099	0.910		
**Intelligence**	**Meta-analysis**	**1220**	**0.020**	**-0.021**	**0.061**	**0.021**	**0.336**	**3.289**	**0.193**

Dimension: Negative symptoms of diminished expressivity									
**PRS**	**Study**	**N**	**Beta**	**L95**	**U95**	**SE**	**P-val**	**Q**	**Q p-val**
Bipolar	CardiffCOGS	697	0.043	-0.034	0.119	0.039	0.273		
Bipolar	Cardiff F-series	383	-0.113	-0.218	-0.007	0.054	0.036		
Bipolar	Cardiff Affected-Sib	140	0.174	-0.033	0.381	0.105	0.099		
**Bipolar**	**Meta-analysis**	**1220**	**0.004**	**-0.055**	**0.064**	**0.030**	**0.882**	**8.391**	**0.015**
Depression	CardiffCOGS	697	-0.024	-0.097	0.050	0.038	0.532		
Depression	Cardiff F-series	383	-0.137	-0.236	-0.038	0.050	0.007		
Depression	Cardiff Affected-Sib	140	0.050	-0.165	0.265	0.108	0.646		
**Depression**	**Meta-analysis**	**1220**	**-0.056**	**-0.113**	**0.001**	**0.029**	**0.054**	**4.283**	**0.117**
Autism	CardiffCOGS	697	-0.041	-0.116	0.034	0.038	0.284		
Autism	Cardiff F-series	383	-0.037	-0.141	0.066	0.052	0.475		
Autism	Cardiff Affected-Sib	140	0.053	-0.166	0.273	0.111	0.631		
**Autism**	**Meta-analysis**	**1220**	**-0.033**	**-0.091**	**0.025**	**0.030**	**0.267**	**0.659**	**0.719**
ADHD	CardiffCOGS	697	-0.041	-0.114	0.032	0.037	0.269		
ADHD	Cardiff F-series	383	-0.020	-0.123	0.084	0.053	0.707		
ADHD	Cardiff Affected-Sib	140	0.190	-0.042	0.422	0.117	0.108		
**ADHD**	**Meta-analysis**	**1220**	**-0.020**	**-0.077**	**0.038**	**0.029**	**0.497**	**3.540**	**0.170**
Intelligence	CardiffCOGS	697	-0.084	-0.160	-0.009	0.038	0.029		
Intelligence	Cardiff F-series	383	-0.009	-0.116	0.097	0.054	0.864		
Intelligence	Cardiff Affected-Sib	140	0.224	0.012	0.436	0.107	0.039		
**Intelligence**	**Meta-analysis**	**1220**	**-0.037**	**-0.096**	**0.022**	**0.030**	**0.224**	**7.716**	**0.021**
Dimension: Disorganised symptoms									
**PRS**	**Study**	**N**	**Beta**	**L95**	**U95**	**SE**	**P-val**	**Q**	**Q p-val**
Bipolar	CardiffCOGS	697	0.060	-0.010	0.129	0.035	0.091		

Bipolar	Cardiff F-series	383	-0.062	-0.172	0.048	0.056	0.268		
Bipolar	Cardiff Affected-Sib	140	-0.007	-0.254	0.240	0.124	0.955		
**Bipolar**	**Meta-analysis**	**1220**	**0.023**	**-0.034**	**0.080**	**0.029**	**0.422**	**3.446**	**0.179**
Depression	CardiffCOGS	697	-0.014	-0.081	0.053	0.034	0.688		
Depression	Cardiff F-series	383	-0.145	-0.248	-0.042	0.052	0.006		
Depression	Cardiff Affected-Sib	140	-0.068	-0.320	0.185	0.127	0.597		
**Depression**	**Meta-analysis**	**1220**	**-0.054**	**-0.108**	**0.001**	**0.028**	**0.055**	**4.414**	**0.110**
Autism	CardiffCOGS	697	0.048	-0.020	0.116	0.035	0.164		
Autism	Cardiff F-series	383	-0.016	-0.123	0.091	0.054	0.770		
Autism	Cardiff Affected-Sib	140	0.056	-0.202	0.314	0.130	0.668		
**Autism**	**Meta-analysis**	**1220**	**0.031**	**-0.025**	**0.087**	**0.029**	**0.277**	**1.028**	**0.598**
ADHD	CardiffCOGS	697	-0.005	-0.071	0.061	0.034	0.883		
ADHD	Cardiff F-series	383	0.019	-0.088	0.126	0.055	0.730		
ADHD	Cardiff Affected-Sib	140	-0.008	-0.284	0.269	0.139	0.956		
**ADHD**	**Meta-analysis**	**1220**	**0.001**	**-0.054**	**0.056**	**0.028**	**0.965**	**0.142**	**0.931**
Intelligence	CardiffCOGS	697	-0.049	-0.118	0.020	0.035	0.160		
Intelligence	Cardiff F-series	383	-0.138	-0.247	-0.028	0.056	0.014		
Intelligence	Cardiff Affected-Sib	140	0.171	-0.082	0.423	0.127	0.183		
**Intelligence**	**Meta-analysis**	**1220**	**-0.062**	**-0.118**	**-0.005**	**0.029**	**0.033**	**5.337**	**0.069**

Association of related PRS with 3-factor dimension scores in each sample and meta-analysis. Columns represent the PRS tested (bipolar disorder, major depression, autism spectrum disorder, ADHD, and intelligence), sample tested (CardiffCOGS, Cardiff F-series, Cardiff Affected-Sib, and meta-analysis), numbers in each sample, beta association value, lower 95% confidence interval of the beta (L95), upper 95% confidence interval of the beta (U95), standard error (SE), association p-value (P-val), Q value and Q p-value (meta-analysis only).

eReferences

1. Deelen P, Bonder MJ, van der Velde KJ, et al. Genotype harmonizer: automatic strand alignment and format conversion for genotype data integration. *BMC Res Notes.* 2014;7:901.
2. McCarthy S, Das S, Kretzschmar W, et al. A reference panel of 64,976 haplotypes for genotype imputation. *Nat Genet.* 2016;48(10):1279-1283.
3. Das S, Forer L, Schonherr S, et al. Next-generation genotype imputation service and methods. *Nature Genetics.* 2016;48(10):1284-1287.
4. Chang CC, Chow CC, Tellier LC, Vattikuti S, Purcell SM, Lee JJ. Second-generation PLINK: rising to the challenge of larger and richer datasets. *GigaScience.* 2015;4:7.
5. Schizophrenia Working Group of the Psychiatric Genomics Consortium. Biological insights from 108 schizophrenia-associated genetic loci. *Nature.* 2014;511(7510):421-427.
6. Bentler PM. Comparative fit indexes in structural models. *Psychol Bull.* 1990;107(2):238-246.
7. Schreiber JB, Nora A, Stage FK, Barlow EA, King J. Reporting structural equation modeling and confirmatory factor analysis results: A review. *Journal of Educational Research.* 2006;99(6):323-337.
8. Hu LT, Bentler PM. Cutoff Criteria for Fit Indexes in Covariance Structure Analysis: Conventional Criteria Versus New Alternatives. *Structural Equation Modeling-a Multidisciplinary Journal.* 1999;6(1):1-55.

1. Deelen P, Bonder MJ, van der Velde KJ, et al. Genotype harmonizer: automatic strand alignment and format conversion for genotype data integration. *BMC Res Notes.* 2014;7:901.
2. McCarthy S, Das S, Kretzschmar W, et al. A reference panel of 64,976 haplotypes for genotype imputation. *Nat Genet.* 2016;48(10):1279-1283.
3. Das S, Forer L, Schonherr S, et al. Next-generation genotype imputation service and methods. *Nature Genetics.* 2016;48(10):1284-1287.
4. Chang CC, Chow CC, Tellier LC, Vattikuti S, Purcell SM, Lee JJ. Second-generation PLINK: rising to the challenge of larger and richer datasets. *GigaScience.* 2015;4:7.
5. Schizophrenia Working Group of the Psychiatric Genomics Consortium. Biological insights from 108 schizophrenia-associated genetic loci. *Nature.* 2014;511(7510):421-427.
